# Evaluation of a *Salmonella* Vectored Vaccine Expressing *Mycobacterium avium* Subsp. *paratuberculosis* Antigens Against Challenge in a Goat Model

**DOI:** 10.1371/journal.pone.0070171

**Published:** 2013-08-09

**Authors:** Syed M. Faisal, Falong Yan, Tsai-Tzu Chen, Nicodemus M. Useh, Shanguang Guo, Weiwei Yan, Shih-Jon Wang, Amy L. Glaser, Sean P. McDonough, Bhupinder Singh, Yung-Fu Chang

**Affiliations:** 1 Department of Population Medicine and Diagnostic Sciences, College of Veterinary Medicine, Cornell University, Ithaca, New York, United States of America; 2 Department of Biomedical Sciences, College of Veterinary Medicine, Cornell University, Ithaca, New York, United States of America; 3 Center for Animal Resources & Education, College of Veterinary Medicine, Cornell University, Ithaca, New York, United States of America; 4 Graduate Institute of Agriculture, National Chiayi University, Chiayi, Taiwan; 5 Department of Bioscience Technology, Chang Jung Christian University, Tainan, Taiwan; Monash University, Australia

## Abstract

Johnes disease (JD), caused by *Mycobacterium avium* subsp *paratuberculosis* (MAP), occurs worldwide as chronic granulomatous enteritis of domestic and wild ruminants. To develop a cost effective vaccine, in a previous study we constructed an attenuated *Salmonella* strain that expressed a fusion product made up of partial fragments of MAP antigens (Ag85A, Ag85B and SOD) that imparted protection against challenge in a mouse model. In the current study we evaluated the differential immune response and protective efficacy of the Sal-Ag vaccine against challenge in a goat model as compared to the live attenuated vaccine MAP316F. PBMCs from goats vaccinated with Sal-Ag and challenged with MAP generated significantly lower levels of IFN-γ, following in vitro stimulation with either Antigen-mix or PPD jhonin, than PBMC from MAP316F vaccinated animals. Flow cytometric analysis showed the increase in IFN-γ correlated with a significantly higher level of proliferation of CD4, CD8 and γδT cells and an increased expression of CD25 and CD45R0 in MAP316F vaccinated animals as compared to control animals. Evaluation of a range of cytokines involved in Th1, Th2, Treg, and Th17 immune responses by quantitative PCR showed low levels of expression of Th1 (IFN-γ, IL-2, IL-12) and proinflammatory cytokines (IL-6, IL-8, IL-18, TNF-α) in the Sal-Ag immunized group. Significant levels of Th2 and anti-inflammatory cytokines transcripts (IL-4, IL-10, IL-13, TGF-β) were expressed but their level was low and with a pattern similar to the control group. Over all, Sal-Ag vaccine imparted partial protection that limited colonization in tissues of some animals upon challenge with wild type MAP but not to the level achieved with MAP316F. In conclusion, the data indicates that Sal-Ag vaccine induced only a low level of protective immunity that failed to limit the colonization of MAP in infected animals. Hence the Sal-Ag vaccine needs further refinement to increase its efficacy.

## Introduction

*Mycobacterium avium* subsp *paratuberculosis* (MAP) causes JD which is characterized by gradual weight loss and diarrhoea due to chronic, progressive, granulomatous enteritis and eventually death [1]. Considerable economic losses occur worldwide due to reduced milk production and to premature culling of diseased animals [2], [3]. Moreover, MAP infection has gained significance because of its suspected involvement in human Crohn's disease [3]–[5]. The available vaccines induce suboptimal levels of protection, associated with adverse side effects like granulomatous lesions and above all interfere with current cellular immune assays for bovine tuberculosis restricting their potential use in many countries [6]–[12]. Thus, the current research priority is to develop potent vaccine candidates that can stimulate protective immunity but do not interfere with tuberculosis control programs. Although several different vaccine constructs have been developed, including live vaccines, subunit vaccines and DNA vaccines, the level of protection that they induce has not exceeded the levels conferred by live attenuated or killed MAP vaccination [13]. Subunit vaccines are a promising strategy and several MAP antigens including Ag85A, Ag85B, and superoxide dismutase (SOD) have imparted protection in murine, caprine and bovine models [14]–[17]. However subunit vaccines are often associated with high cost and require strong adjuvants, which cause toxicity and local inflammatory reactions thus limiting their application in the field. Moreover, these vaccines failed to prevent MAP shedding in feces of infected animals. Due to these limitations there is considerable interest in developing alternate vaccination strategies that are cost effective, easily administered and can be applied as prophylaxis against JD. Attenuated *Salmonella* strains hold great promise as live vectors for presentation of foreign antigens from unrelated bacterial, viral and parasitic pathogens to the immune system [18]. This strategy has already been shown to induce protective immune responses against various infections in a variety of animal models. Vaccination with attenuated *Salmonella* vector expressing various antigens of HIV and *Mycobacterium* resulted in the generation of both *Salmonella* and heterologous antigen specific humoral and cellular immune responses, normally biased towards TH1 [19]–[25]. In the previous study we demonstrated that immunization with attenuated *Salmonella* expressing a fusion product of partial fragments of predominant and protective MAP antigens (Sal-Ag) imparted significant protection against challenge in mice [26]. Encouraged by these results, we wanted to test whether this strategy could impart protection against challenge in a natural host. In the present study we evaluated the differential immune response and protective efficacy of an attenuated *Salmonella* vaccine candidate that expresses partial fragments of predominant and protective MAP antigens as a fusion product (Sal-Ag) against challenge in a goat model as compared to the live attenuated vaccine, MAP316F.

## Materials and Methods

### Animals

A total of 24 neutered male or female goats between 9–12 weeks old (uniform Boer from one dairy breed and randomized for ages), were obtained from a local farm. Fecal samples taken from the goats before immunization were negative for MAP and other pathogens, both by culture and by PCR for the IS*900* gene. The goats were injected i.d. with 0.1 ml of avian PPD (0.5 mg/ml) four days before vaccination and one week before euthanasia and the increase in skin fold thickness was measured after 72 hrs by caliper. All the goats were negative on the intradermal skin test (IDT). **This study was conducted in compliance with the regulations, policies, and principles of the Animal Welfare Act, the Public Health Service Policy on Humane Care and Use of Laboratory Animals used in testing, research, and training, the NIH Guide for the care and use of laboratory animals.** **The protocol was approved by the Committee on the Ethics of Animal Experiments of the Cornell University (IACUC Number: 2009–0115). All efforts were made to minimize animal suffering.**

### Bacterial strains

MAP 66115-98, a clinical isolate, was used to challenge the goats after immunization [15], [16]. This strain is IS*900* positive and mycobactin dependent. MAP 66115-98 was grown in 7H9 medium supplemented with 10% oleic acid–albumin–dextrose–catalase (Becton Dickinson Co., Sparks, MD) and mycobactin J (Allied Monitor, Inc., Fayette, MO). After culturing for 8 weeks, the organisms were harvested by centrifugation at 4000×*g* for 10 min and washed twice with phosphate-buffered saline (10 mM PBS; pH 7.2). The organisms were diluted in PBS to the required concentration for challenge studies. MAP316F, a live attenuated vaccine, was obtained from Dr. R. A. Juste, NIEKER, Spain as a gift.

### *Salmonella* mutant construction

The virulence genes *yejE* and *ssaV* were knocked out from *S. enterica* serovar Typhimurium (*S.* Typhimurium) by one using the one step gene inactivation by PCR technique as previously described [26]. Briefly, the Cat sequence (chloramphenicol cassette) from plasmid pKD3 was PCR amplified using primers that contained flanking sequences of the gene (*yejE*) and the PCR product was purified. *S.* Typhimurium transformed with a Red helper plasmid (pKD46) was made electrocompetent, electroporated with PCR product and grown to select for Cm^R^ or Km^R^ transformants. Cm^R^ mutants were transformed with pCP20 (an ampicillin and CmR plasmid that shows temperature-sensitive replication and thermal induction of FLP synthesis) and ampicillin-resistant transformants were selected at 30°C followed by 43°C and then tested for loss of all antibiotic resistance. The majority lost the FRT-flanked resistance gene and the FLP helper plasmid simultaneously. The *S.* Typhiumurim (Δ*yejE*) mutant thus obtained was then targeted to knock out the *ssav* gene using a similar procedure to get the *S.* Typhiumurim (Δ*yejE*; Δ*ssaV*) mutant. The mutation in S. Typhimurium was first confirmed by PCR using primers for specific genes and then by sequencing. Attenuation was confirmed by injecting both the mutant and salmonella WT strains (10^9^ CFU in 500 ul of PBS) intraperitoneally in C57/BL6 mice and measuring the bacterial load in the liver and spleen at 12 and 16 weeks as previously described [23].

### Cloning and Expression of MAP antigens into the *Salmonella* mutant

A portion of the amino terminus (N terminal 1–104 amino acids) of the Type III secretion system (T3SS) effector protein gene with its promotor, called SopE-104, was PCR amplified from the plasmid pSB1120 and cloned into a pSU39 expression vector to yield pSU39sopE104. Various portions from the N and C terminal regions of genes coding for MAP antigens (Ag85A, Ag85B,SOD) were PCR amplified, cloned into the pSU39sopE104 vector, transformed into *E. coli* and selected on kanamycin plates. The cloned genes were transformed into attenuated *S.* Typhimurium and protein was analyzed both from cell pellets and culture supernatant [27]. The expressed and secreted proteins were analyzed by western blot using anti-HA mouse IgG following standard procedures.

### Immunization

The goats were divided into four groups namely A (PBS), B (MAP316F), C (Sal-Vector) and D (Sal-Ag), with six animals in each and immunized, bled or challenged according to the schedule presented in Table 1. The animals were immunized subcutaneously in the lower left side of the neck. Three weeks after the primary immunization, the goats were boosted with the same regimen. All the animals were euthanized 24 weeks post challenge.

**Table 1:** Schedule for vaccination and challenge of goats.

Groups	Vaccine	Number of goats	Goat ID	Dose	Route
**A**	PBS	6	2194, 2198, 2199, 2200, 2201, 2202	1ml	SC*
**B**	MAP316F	6	2220, 2222, 2224, 2226, 2230, 2234	5×10^8^CFU	SC*
**C**	Sal-Vector	6	2183, 2184, 2189, 2190, 2191, 2192	5×10^8^CFU I ml PBS	SC
**D**	Sal-Ag	6	2236, 2237, 2238, 2239, 2241, 2243	5×10^8^CFU	SC

The goats were vaccinated and then boosted at 3 week. All the animals were challenged orally for 7 days with 5×10^8^ CFU MAP in 10 ml PBS. The animals were bled (10 ml, jugular vein) and feces (10 g) were collected at 0 wk, 3 wk, 6 wk, 10 wk, 14 wk, 18 wk, 22 wk, 26 wk and 30 wk.All the animals were necropsied at week 30.

* SC- Subcutaneous, CFU- colony forming units.

### Challenge

Three weeks after the booster, all 24 goats were challenged orally with 5×10^8^ CFU of MAP 66115-98 in 10 ml PBS for 7 consecutive days. Fecal cultures were performed on each animal on days 2, 4 and 6 after each challenge and then once every month.

### Antibody response

Sera were harvested from blood collected at various time points and indirect ELISA was performed to detect antibodies (IgG), as previously described [28], [29]. Briefly, 96-well flat bottom plates (Nunc Maxisorp) were coated with 100 μl of 15 µg/ml of Ag-mix (5 µg/ml of each Ag85A, Ag85B and SOD) or 10 μg/ml johnin purified protein derivative (PPDj; DBL, National Veterinary Services Laboratory, Ames, IA) and kept at 4°C overnight in a humidified atmosphere. The plates were washed three times with PBS containing 0.05% Tween 20 (PBST) and blocked with 5% skim milk in PBST at 37°C for 1 h. After washing with PBST, 100 μl of diluted serum (1:200) were added to the wells and the plates were incubated at 37°C for 2 h. After washing, 100 µl of rabbit anti-goat IgG-conjugated with horseradish peroxidase (1:3000) was added and plates were further incubated at 37°C for 45–60 min. The plates were washed three times in PBST, 100 μl of TMB substrate was added to each well and plates were incubated in the dark at room temperature for 20 min. The enzymatic reaction was stopped by the addition of 1 M H_2_SO_4_, and the optical density (OD) was read at 450 nm using an EL_X_ 808 Ultra microplate reader (Bio-Tek Instruments, Inc., Winooski, VT). Suitable positive and negative sera and antigen and antibody controls were included in each plate. The results are expressed as ELISA units  =  (sample OD−negative control OD) ×100.

### Isolation and culture of peripheral blood mononuclear cells (PBMC)

PBMCs were isolated from the experimental goats as described previously [16], [29]. Briefly, 10–15 ml of peripheral blood was collected from the jugular vein into EDTA vacutainer tubes (Becton Dickinson and Co., Franklin Lakes, NJ). Blood was centrifuged and after removing the buffy coat, lymphocytes were isolated by differential centrifugation using Histopaque 1.077 (Sigma–Aldrich, St. Louis, MO). The mononuclear cells were washed three times with PBS (pH 7.2).Washed cell pellets were resuspended in PBS and counted after staining with 0.4% trypan blue. Cell viability determined by trypan blue exclusion, was consistently >90%. The lymphocytes were resuspended in RPMI-1640 medium containing 10% fetal bovine serum (Gibco, Grand Island, NY), 2 mM l-glutamine, 100 mM HEPES, 100 IU/ml of penicillin, 100 µg/ml of streptomycin and 50 µg/ml of gentamycin (Gibco), to a final concentration of 2×10^6^ viable cells/ml. The cells were then seeded (200 µl/well) onto 96-well round or flat bottom plates, depending on the type of experiment.

### Lymphoproliferation

Lymphocyte proliferation assays were performed as previously described [16], [29]. Briefly, 2×10^5^ PBMCs in 96-well flat bottom plates were stimulated with Ag-mix (15 μg/ml) or 10 μg/ml PPDj for 72 h. The stimulating doses of both PPDj and Ag-mix were selected based on previous studies [16] and were used for stimulation of samples from all groups. However Ag mix was used to analyze samples from vector and Sal-Ag groups whereas PPDj was used to analyze samples from PBS and MAP316F groups. DNA synthesis in stimulated and un-stimulated control cells was measured by the incorporation of bromodeoxyuridine (BrdU) by Cell proliferation ELISA, BrdU colorimetric kit (Roche Diagnostics, Indianapolis, IN) as per the manufacturer's protocol. Briefly, the cells were labeled for 2 h with 10 μl of BrdU labeling solution. The peroxidase-conjugated anti-BrdU antibody was added and incubated for 90 min. This was followed by the addition of the enzyme substrate solution and incubation at room temperature for 15 min. The enzymatic reaction was stopped by the addition of 1 M H_2_SO_4_, and the optical density (OD) was read at 450 nm using an EL_X_ 808 Ultra microplate reader (Bio-Tek Instruments, Inc., Winooski, VT). The tests were run in triplicate, and the results are expressed as the average stimulation index (SI), calculated as the ratio between the mean OD of cells cultured with the PPDj and the mean OD of cells cultured without PPDj.

### Cytokine analysis by RT-PCR

To compare the expression levels of selected immune response genes of PBMCs isolated from goats at various time points, 10^7^ PBMCs in 6-well flat bottom plates were stimulated with Ag-mix (15 μg/ml) or 10 μg/ml PPDj as described previously [29]–[31,31]. After 3 days of incubation, total RNA was extracted from the pooled PBMCs of both stimulated and unstimulated wells. Total RNA (3 μg) was reverse-transcribed using oligo(dT) primers and the Superscript III First-Strand Synthesis System (Invitrogen) according to the manufacturer's specifications. The selected immune response genes, forward and reverse primers, product lengths and GenBank accession numbers of the sequences used to design the primers are listed in Table 2. All primer pairs were designed to target areas with minimal secondary structure, to work at an annealing temperature of 60°C and, where feasible, to span two exons. Real-time PCR was performed on an ABI 7500 FAST sequence detection system (Applied Biosystems) by using Power SYBR Green Master Mix (Invitrogen) in a 20 µl reaction volume. Primers were used at a final concentration of 200 nM. Reactions were performed in 96-well MicroAmp Fast optical plates (Applied Biosystems) sealed with optical adhesive covers (Applied Biosystems).Thermal cycling conditions consisted of enzyme activation at 95°C for 15 min, followed by 40 cycles of denaturation at 95°C for 15 s and annealing and extension at 60°C for 60 s. No-template controls (NTC) were included for each target on each plate (data not shown). Post-PCR dissociation melting curves were determined for every reaction to confirm specificity and melting temperature of the amplification products (data not shown). The resulting data were analyzed by the 2^−ddCt^ method with GAPDH as the internal control and unstimulated sample as the calibrator using the 7500 Fast System SDS Software version 1.4 (Applied Biosystems). The data have been grouped to compare the cytokine profiles considered to define Th1, Th2, Th17, and regulatory T cells (Treg). The tests were run in triplicates.

**Table 2:** Sequence of primers used in the study.

Gene	Primer	Sequence	Amplicon (bp)	Gene Accession number
**IL-2**	F	TGAAAGAAGTGAAGTCATTGCTGC	138	NM_001009806
	R	GATGTTTCAATTCTGTAGCGTTAACC		
**IL-4**	F	ACCTGTTCTGTGAATGAAGCCAA	79	NM_001009313
	R	CCCTCATAATAGTCTTTAGCCTTTCC		
**IL-6**	F	CGCTCCCATGATTGTGGTAGTT	64	NM_001009392
	R	GCCCAGTGGACAGGTTTCTG		
**IL-8**	F	CGAAAAGTGGGTGCAGAAGGT	80	NM_001009401
	R	GGTTGTTTTTTCTTTTTCATGGA		
**IL-10**	F	AGCAAGGCGGTGGAGCAG	90	NM_001009327
	R	GATGAAGATGTCAAACTCACTCATGG		
**IL-12**	F	GCTGGGAGTACCCTGACACG	127	NM_001009438
	R	GTGACTTTGGCTGAGGTTTGGTC		
**IL-13**	F	CAGTGTCATCCACAGGACCAAG	90	NM_001082594
	R	TCTCGGACGTACTCACTGGAAAC		
**IL-17**	F	CATCATCCCACAGAGTCCAGG	201	AF412040
	R	CACTTGGCCTCCCAGATCAC		
**IL-18**	F	ACTGTTCAGATAATGCACCCCAG	100	NM_001009263
	R	TTCTTACACTGCACAGAGATGGTTAC		
**IL-23**	F	CCTCCTTCTCCGTCTCAAGATC	131	XM_588269
	R	CGGAGGTCTGGGTGTCATCCT		
**IL-1b**	F	CCTAACTGGTACATCAGCACTTCTCA	95	NM_001009465
	R	TCCATTCTGAAGTCAGCACTTCTCA		
**IFN-g**	F	GATAACCAGGTCATTCAAAGGAGC	124	NM_001009803
	R	GATCATCCACCGGAATTTGAATC		
**TNF-a**	F	GCCCTGGTACGAACCCATCTA	82	NM_001024860
	R	CGGCAGGTTGATCTCAGCAC		
**TGF-b**	F	CTGAGCCAGAGGCGGACTAC	63	NM_001009400
	R	TGCCGTATTCCACCATTAGCA		
**GAPDH**	F	GAGAAGGCTGGGGCTCACC	129	AF030943
	R	GCTGACAATCTTGAGGGTATTGTT		

### IFN-γ assay

A total of 2×10^5^ PBMCs in each well of 96-well flat bottom plates were stimulated with Ag-mix (15 μg/ml) or 10 μg/ml of PPDj for 72 h. IFN-γ levels were measured in the culture supernatants using a monoclonal antibody-based sandwich enzyme immunoassay (BOVIGAM; Biocor Animal Health, Omaha, NE), as per the manufacturer's instructions [16]. The plates were read at 450 nm using an ELx 808 Ultra microplate reader (Bio-Tek Instruments, Inc.). Results were considered positive (OD > positive control) or negative (OD < positive control), relative to the cutoff values suggested by the manufacturer and expressed as mean OD in stimulated wells minus OD in un-stimulated wells. The tests were run in triplicates.

### Flow Cytometric analysis

Flow cytometric (FC) analysis of PBMCs isolated from animals bled at different time points was performed as described previously [31]. PBMCs (10^7^) from the different groups were cultured for 6 days with/without Ag-mix (15 μg/ml) or PPDj (10 µg/ml) in 6 well tissue culture plates. Cells were recovered and 10^6^ cells were labeled with goat-specific monoclonal antibodies: (CD2-MUC2A-IgG2a; CD4-GC1A1-IgG2a; CD8-7C2B-IgG2_a_; CD25-CACT116A-IgG_1_; CD45R0-ILA116A-IgG3; γδTCR-GB21A-IgG2b (Washington State University, Monoclonal Antibody Center, Pullman WA) as previously described. The cells were stained with three color combination as CD4/CD25/CD45R0, CD8/CD25/CD45R0 or CD2/CD25/γδTcells to analyze the activation status of memory CD4, CD8 and γδT cells. Briefly, the cells were washed three times with FC buffer (20% acid-citrate dextrose, 4% horse serum) and incubated with a cocktail of the primary antibodies (15 µg/ml, previously titrated for optimum reactivity) for 15 min at 4°C. The lymphocytes were then washed three times and incubated for an additional 15 min at 4°C in cocktails of isotype-specific secondary antibodies viz. PE-Cy5.5/PE-Cy5/FITC for analyzing CD4/CD25 or CD4/CD45R0 T cells and CD8/CD25 or CD8/CD45R0 T cells and PE-Cy5.5/PE-Cy5/PE for analyzing CD2/CD25/γδT cells (Invitrogen; Southern Biotechnology Associates Birmingham, AL). Cells were washed three times and suspended in 200 µl of FC buffer. A total of 50,000 events were acquired on a BD LSR II housed in the Biomedical Sciences Flow Cytometer Core Laboratory at Cornell University. All the data were analyzed by BD FACS Diva software (BD). The percent activated (CD25+) and memory phenotype (CD45R0+) of CD4 or CD8 T cells in the total PBMC pool was determined by using electronic gates to isolate CD4 and CD8 populations for analysis. The tests were run in triplicates.

### Necropsy

All animals were euthanized using a captive bolt stun gun. After exsanguination, theintestines were removed from below the abomasum to the rectum and laid out to expose the jejunum, ileum, cecum and lymph nodes. Samples were taken from serial sections of the mesenteric lymph nodes (MLN), the ileocecal lymph node (ICLN), descending duodenum (Dd), proximal, middle and distal portions of jejunum (JP,JM,JD), proximal, middle and distal portions of ileum (IP,IM,ID), ileocecal orifice (ICO) and cecum (C).

### Fecal and organ culture of MAP and MAP PCR

Following challenge, attempts were made to isolate MAP organisms from feces using Herald's egg yolk (HEY) medium (Becton, Dickinson and Co., Sparks, MD) [15], [32]. Fecal samples were collected from all animals at 2, 4, 6, 8 and 10 days after challenge, and every month thereafter for MAP isolation. Similarly, 9 tissue samples collected from each of the 24 animals at necropsy were also tested for MAP by culture. Cultures were performed by the Bacteriology section at the Cornell Animal Health Diagnostic Center. Briefly 1 g of tissue or fecal samples were homogenized separately in 15 ml of PBS in whirl pack bags using stomacher for 5 minutes. 10 ml aliquot was removed from the bag and 5 ml was added in each 25 ml brain heart infusion broth supplemented with hexadecylpyridinium chloride monohydrate (BHI/HPC) tubes and incubated at 35°C for 12–16 hrs. Tubes were centrifuged (3000 rpm for 20 min) and supernatant was decanted. The pellet was suspended in 1 ml antibiotic brew (BHI broth containing 100 µg/ml nalidixic acid, 100 µg/ml vancomycin and 50 µg/ml amphotericin B) and further incubated for 18hrs at 35°C. An aliquot (100 ul) was inoculated in three HEY slants with or without Mycobactin J and incubated for 8 weeks at 35°C. The colonies were counted and data was presented as + (less than 50 colonies), ++ (50–300 colonies), +++ (>300 colonies) or – (no colony detected). PCR was performed by the Molecular Diagnostic section at the Cornell Animal Health Diagnostic Center. DNA was extracted from glass bead-disrupted lysates using an automated 96 well nucleic acid, magnetic bead-based purification system (Kingfisher 96, Thermo Fisher Scientific Inc., Pittsburg, PA) and DNA was amplified using a commercial TaqMan MAP assay reagents (MAP reagents, Life Technologies, Grand Island, NY). All individuals engaged in MAP testing were blinded to the treatment group. The tests were run in triplicates.

### Gross pathology and histopathological examination

All the goats were euthanized 30 weeks after primary vaccination and necropsied. A total of 9 tissue samples from each animal, which included mesenteric lymph nodes (3 sites), ileocecal lymph node, descending duodenum, jejunum (three sites of approximately equal intervals from proximal to distal end), ileum (two sites at proximal end, two sites at mid ileum and two sites at distal end), ileocecal orifice, and cecum, were collected at the time of necropsy. Collected tissues were fixed by immersion in 10% neutral buffered formalin, embedded in paraffin wax, sectioned at 4 µm and stained with hematoxylin and eosin (H&E) and Ziehl-Neelson for acid-fast bacteria using conventional histological methods. Sections were examined by a board certified veterinary pathologist (SPM), who was blinded to the treatment group.

### Statistical analysis

The data were statistically analyzed with the Excel software. Differences between groups were analyzed with one-way analysis of variance (ANOVA) followed by Tukey–Kramer multiple comparison or Student's *t*-test. The numbers of MAP culture positive animals between groups were compared by Fisher's exact test. For all tests, differences were considered significant when a probability value of <0.05 was obtained.

## Results

### Humoral immune response

**Figure 1:**
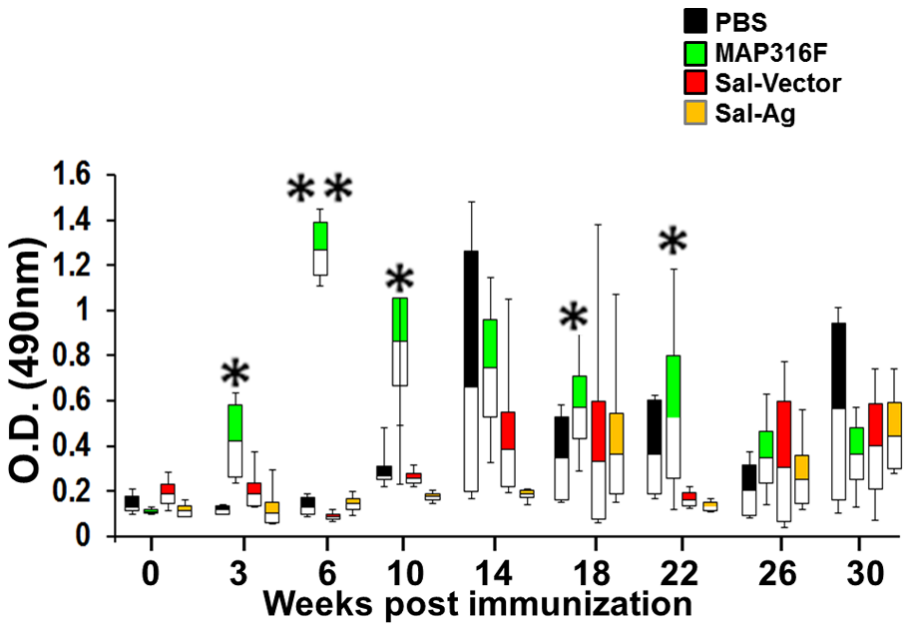
Antibody response. Blood collected at various time points was allowed to clot and serum collected was analyzed for total IgG against Ags (85A, 85B, SOD) or jhonin PPD at serum dilution of 1:200 using indirect ELISA as described in material and methods. The response is measured in individual goats and data is represented in the form of whisker-box plots (PBS Vs 316F * indicates p<0.05 ** indicates p<0.01).

The antibody response varied at various time points within groups and some animals did not produce significant antibody levels. Significant levels of antibodies were detected in MAP316F vaccinated goats three weeks post immunization (p<0.05), which was enhanced (P<0.01) after booster (before challenge). In contrast, Sal-Ag induced only low or undetectable levels of antibodies, similar to the control groups (Fig. 1). The level of antibodies sharply declined after challenge in the MAP316F vaccinated group but was still significantly higher in a greater proportion of animals as compared to the Sal-Ag group. Antibodies were detected at the time of necropsy (week 30) in all groups but the levels were similar. Control animals (PBS and Sal-Vector) generated antibodies after challenge, but the levels were significantly lower than those of the MAP316F vaccinated group and equivalent to the level detected in Sal-Ag immunized animals.

### IFN-γ response

**Figure 2:**
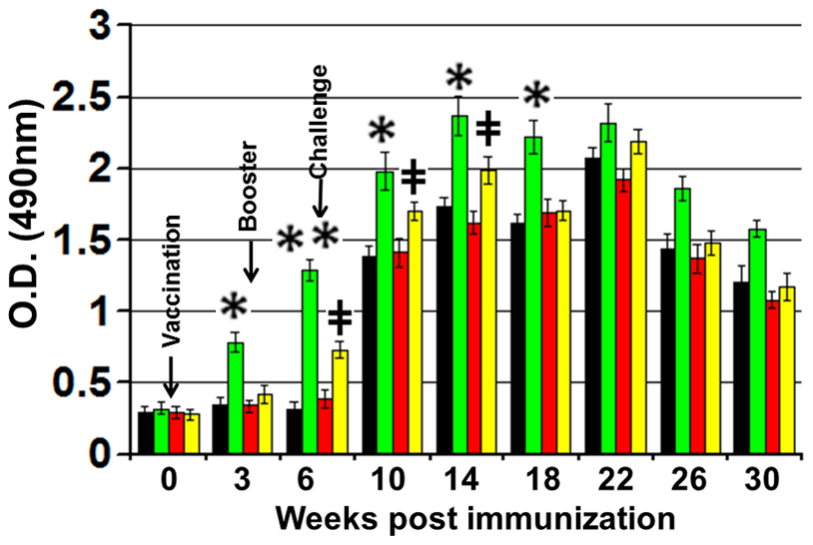
IFN-γ response. PBMCs isolated from animals bled at various time points were stimulated with Ag-mix (15 μg/ml) or PPDj (10 µg/ml) in 200ul RPMI for 72 hrs at 37C in a humidified atmosphere supplemented with 5% CO2. IFN-γ levels in the tissue culture supernatant were determined with a BOVIGAM kit following the manufacturer's protocol. Results are expressed as OD values and error bars indicate 1 standard deviation from the mean. (PBS Vs 316F *indicates p<0.05 ** indicates p<0.01, PBS Vs Sal-Ag **‡** indicates p<0.05 **‡‡** indicates p<0.01).

Significant levels of IFN-γ (p<0.01) were generated in stimulated PBMCs obtained from animals immunized with MAP316F, which was enhanced after booster (Fig. 2). The response was further enhanced after challenge and was significantly higher than control groups (PBS and Sal-vector). Sal-Ag immunized animals generated a low level of IFN-γ that was slightly enhanced after challenge and the rate of increase of IFN-γ slowed after 10 weeks and declined after 22 weeks. Moreover, the response was significantly higher than PBS or vector control group at 6, 10, and 14 week (p<0.05) but there was no augmentation on production of IFN-γ upon in-vitro stimulation of PBMCs with Ag-mix obtained from Sal-Ag vaccinated group at other time points (Fig. 2).

### T cell response

**Figure 3:**
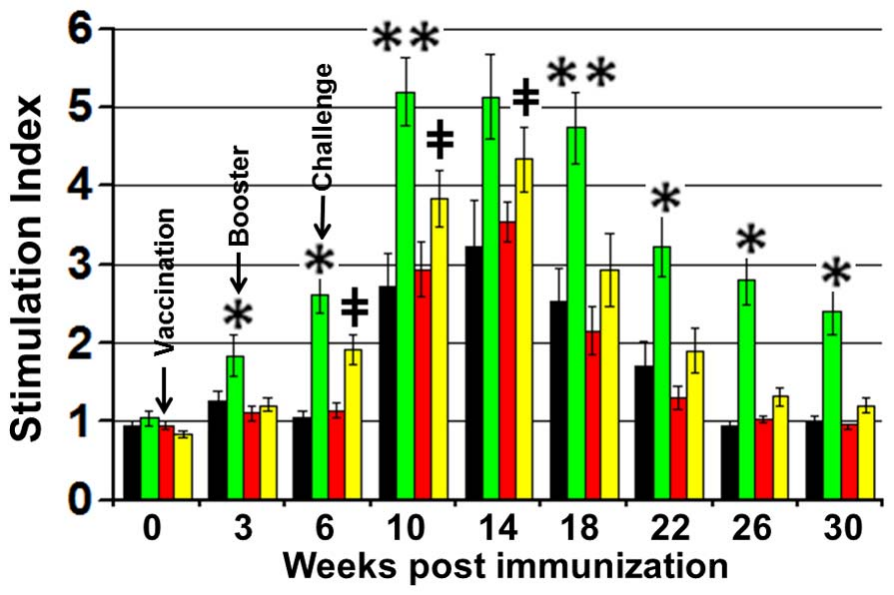
Lymphoproliferation. PBMCs isolated from animals bled at various time points were stimulated with/without Ag-mix (15 μg/ml) or PPDj (10 µg/ml) in 200 ul RPMI for 72 hrs at 37C in a humidified atmosphere supplemented with 5% CO2. The proliferative response was measured by cell proliferation ELISA, BrdU colorimetric kit (Roche Diagnostics, Indianapolis, IN) as per the manufacturer's protocol. The results are expressed as stimulation index (SI), and the error bars indicate 1 standard deviation from the mean. (PBS Vs 316F *indicates p<0.05 ** indicates p<0.01, PBS Vs Sal-Ag **‡** indicates p<0.05 **‡‡** indicates p<0.01).

**Figure 4:**
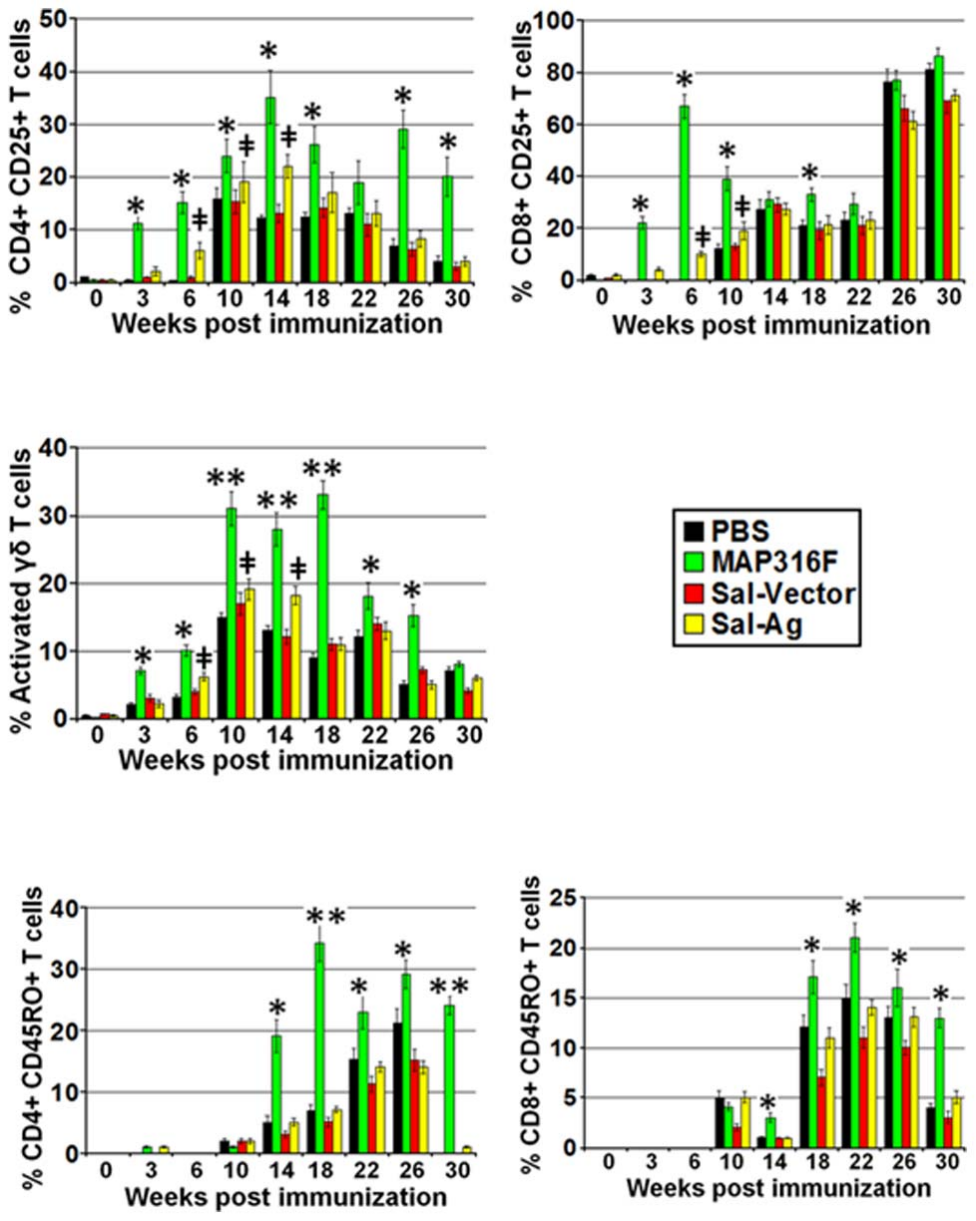
T cell analysis. Comparison of activation and memory status of T cells in PBMCs isolated from animals bled at various time points after immunization, simulated with/without Ag-mix (15 μg/ml) or PPDj (10 µg/ml) for 6 days and subjected to FACS analysis. The data was analyzed by BD FACS Diva software. The data is presented in bar graph form for simplicity. Analysis of activation status (CD25 expression) of (A) CD4 T, (B) CD8 T and (C) γδ T cells at various time points. Analysis of Effector memory phenotype (CD45RO expression) of (D) CD4 and (E) CD8 T cells at various time points. (PBS Vs 316F *indicates p<0.05** indicates p<0.01, PBS Vs Sal-Ag **‡** indicates p<0.05 **‡‡** indicates p<0.01).

A significant proliferative response was detected in PBMCs isolated from MAP316F immunized group, which increased sharply after both booster and challenge and was significantly higher than other groups. A low level of proliferation was observed after booster in the Sal-Ag group, which was significantly enhanced after challenge and the proliferative capacity was significantly higher (p<0.05) at week 6, 10and 14 than PBMCs obtained from PBS or Sal-vector immunized group. (Fig.3). Analysis of activation status of CD4, CD8 and γδT cells by FC analysis at various time points showed that low levels of activated CD4 T cells expressing CD25 were detected in the Sal-Ag group after booster, which was significantly enhanced after challenge. However, MAP316F generated a significantly higher level of activated CD4 T cells that was maintained after challenge (Fig. 4A). A similar pattern was observed for CD25+ CD8+ T cells in the Sal-Ag group (Fig. 4B). The activation status of CD4 and CD8 T cells in PBMCs obtained from Sal-Ag group varied at various time points but the level remained significantly low as compared to MAP316F during the course of the study. Animals immunized with Sal-Ag generated significantly lower levels of activated (CD25 expression) γδT cells than MAP316F which was equivalent to PBS and vector control profiles at all-time points and there was a sharp decline in the number of these cells at 14 week post vaccination (Fig. 4C). The FC analysis further revealed that a significant level of memory response was observed only in MAP316F. Both CD4 and CD8 T cells expressing CD45R0 (effector memory phenotype) were detected 8weeks after challenge in this group and varied at different time points. In contrast, these cells were detected at low levels in Sal-Ag immunized group at few time points after challenge but there was no significant difference from control animals immunized with PBS or vector (Fig. 4D and E).

### Cytokine response

**Figure 5:**
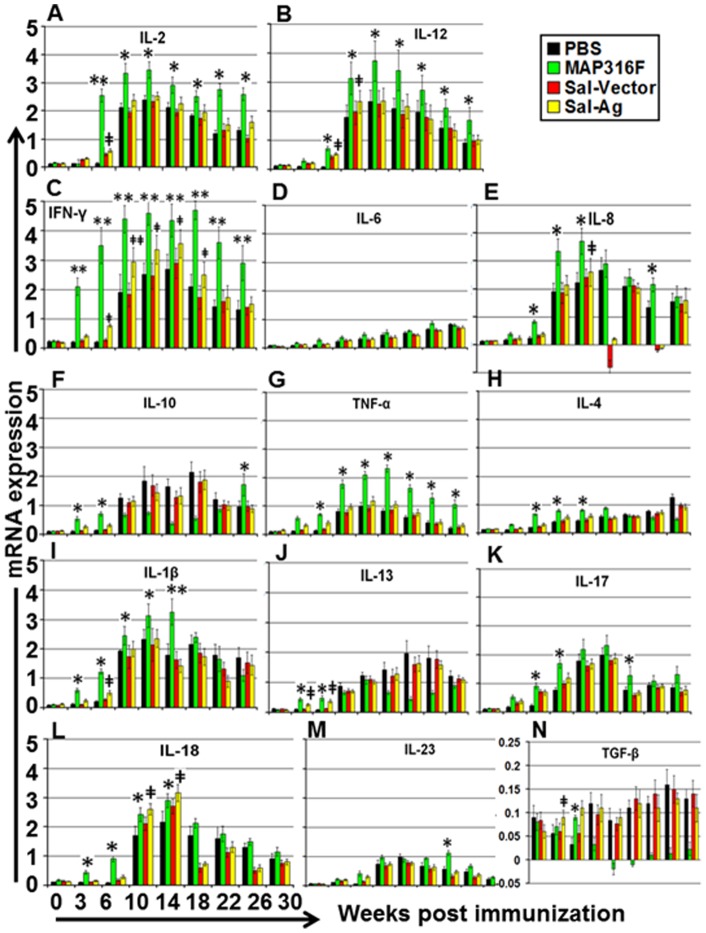
Cytokine analysis by RT-PCR. Relative transcription of Th1, Th2, pro-inflammatory, anti-inflammatory, Th17 and regulatory cytokines in PBMCs isolated from animals bled at various time points after immunization and stimulated with/without Ag-mix (15 μg/ml) or PPDj (10 µg/ml) for 3 days was measured by RT-PCR. The relative transcription was calculated using the value of unstimulated cells as the calibrator with the housekeeping gene GAPDH as an internal control. Data are presented as the relative mRNA expression (mean fold change) and the mean value of each group. The error bars indicate standard deviation from the mean. (PBS Vs 316F *indicates p<0.05 ** indicates p<0.01, PBS Vs Sal-Ag **‡** indicates p<0.05 **‡‡** indicates p<0.01).

MAP316F induced significant upregulation of Th1 cytokines (IFN-γ, IL-2, IL-12 and TNF-α) after vaccination, which was enhanced and maintained at various time points post challenge (Fig. 5). In contrast Sal-Ag induced low levels of these cytokines similar to the pattern observed in the control groups and the level declined sharply 8 weeks post challenge. The levels of proinflammatory cytokines (IL-6, IL-8, IL-18, IL-1β) were significantly higher in MAP316F after challenge whereas Sal-Ag induction of these cytokines was similar to the levels of the control groups (PBS and Sal-Vector) except at week 10 and 14 where induction of IL-18 was significantly higher and at week 18 and 26 where IL-8 and IL-18 levels downregulated. (Fig. 5). Th2 and anti-inflammatory cytokines (IL-4, IL-10, IL-13) were expressed at low levels in all groups after immunization. IL-4 and IL-10 induction was significantly higher in MAP316F than Sal-Ag and control groups at earlier time points (week 6, 10 and 14) and they were down regulated after challenge except at week 30. IL-13 expression was also induced in similar pattern (Fig. 5). Cytokines secreted by Th17 cells, IL-17 and IL-23, were also up-regulated in all groups which was significantly higher in MAP316F, however there was sharp decline in these cytokines 8weeks post challenge in Sal-Ag, similar to control groups.(Fig. 5). There was low level of expression of TGF-β in MAP316F which was downregulated after challenge whereas Sal-Ag and control group induced this cytokine with a similar pattern.

### Protective efficacy of Sal-Ag vaccine against challenge in goats

The protective efficacy was evaluated by assessing MAP burden in various tissues. Culture results shows that all the control animals immunized with PBS had almost all the tissues culture positive and had a higher bacterial load [moderate (>50 CFU) to heavy (>300 CFU)]. Animals immunized with Sal-Vector reduced the bacterial burden in some tissues, indicating non-specific protection but the difference was not significant as compared to PBS control animals. Animals immunized with MAP316F showed significant protection as bacteria were recovered from a few but not all tissues (Table 3). These tissues had moderate to low bacterial loads (< or >50 CFU) while most of them were culture negative. Most importantly, the MAP316F vaccine was not able to impart sterilizing immunity. Animals immunized with Sal-Ag vaccine also showed a reduced bacterial load in some tissues and some were negative, which indicates that Sal-Ag imparted partial protection which was slightly better than Sal-Vector but not to the level achieved with MAP316F. The bacterial load in tissues from both control and vaccinated groups were further confirmed by PCR and it validated the culture results in most tissues except in a few cases where MAP was not detected in culture but was detected by PCR. The protective efficacy was further demonstrated by bacterial shedding where control animals continued high to moderate levels of shedding and MAP316F vaccinated animals showed reduced bacterial shedding, while Sal-Vector and Sal-Ag had low to moderate intermittent shedding at various time points (Table 4). Histopathological examination revealed no lesions in any tissue in any group.

**Table 3:** MAP burden in goat tissues after necropsy.

Groups	GoatID	Tissues																	
		MLN		ICLN		Dd		J		IP		IM		ID		ICO		C	
		CT	PCR	CT	PCR	CT	PCR	CT	PCR	CT	PCR	CT	PCR	CT	PCR	CT	PCR	CT	PCR
**A PBS**	2194	**++**	**++**	**++**	**+++**	**++**	**+++**	**++**	**+++**	**++**	**+++**	**++**	**+++**	**++**	**++**	**++**	**+++**	**++**	**+++**
	2198	**+**	**++**	**−**	**+**	**++**	**+++**	**++**	**+++**	**++**	**+++**	**++**	**+++**	**++**	**++**	**++**	**+++**	**++**	**+++**
	2199	**−**	**+**	**++**	**+++**	**−**	**+**	**++**	**+++**	**++**	**+++**	**++**	**+++**	**++**	**++**	**++**	**+++**	**++**	**+++**
	2200	**++**	**++**	**++**	**+++**	**++**	**+++**	**++**	**+++**	**−**	**−**	**++**	**+++**	**++**	**++**	**−**	**+**	**+**	**+++**
	2201	**++**	**++**	**−**	**−**	**++**	**+++**	**++**	**+++**	**++**	**+++**	**++**	**+++**	**++**	**++**	**++**	**++**	**++**	**+++**
	2202	**++**	**++**	**++**	**+++**	**++**	**+++**	**++**	**+++**	**++**	**+++**	**++**	**+++**	**++**	**++**	**+**	**+**	**++**	**+++**
**B MAP316F**	2220	**−**	**−**	**++**	**++**	**−**	**−**	**+**	**+**	**−**	**−**	**−**	**−**	**+**	**+**	**−**	**−**	**−**	**−**
	2222	**−**	**−**	**−**	**−**	**+**	**+**	**−**	**−**	**−**	**−**	**+**	**+**	**−**	**−**	**−**	**−**	**−**	**−**
	2224	**++**	**++**	**−**	**−**	**+**	**+**	**−**	**−**	**++**	**++**	**−**	**−**	**−**	**−**	**+**	**++**	**++**	**++**
	2226	**−**	**−**	**+**	**+**	**−**	**−**	**+**	**+**	**−**	**−**	**−**	**−**	**+**	**+**	**−**	**−**	**−**	**−**
	2230	**−**	**−**	**−**	**−**	**++**	**++**	**−**	**−**	**−**	**+**	**+**	**−**	**−**	**+**	**+**	**−**	**++**	**++**
	2234	**++**	**++**	**−**	**+**	**−**	**−**	**++**	**++**	**−**	**−**	**−**	**−**	**+**	**+**	**−**	**−**	**++**	**+++**
**C Sal-Vector**	2183	**+**	**++**	**+**	**+**	**−**	**−**	**+**	**+**	**−**	**−**	**−**	**−**	**+**	**+**	**−**	**+**	**++**	**+++**
	2184	**++**	**++**	**−**	**+**	**++**	**+++**	**++**	**+++**	**++**	**+++**	**++**	**++**	**++**	**++**	**++**	**+++**	**++**	**+++**
	2189	**+**	**+**	**++**	**++**	**+**	**+**	**−**	**−**	**++**	**++**	**−**	**+**	**++**	**++**	**++**	**++**	**++**	**+++**
	2190	**++**	**++**	**++**	**++**	**++**	**+++**	**++**	**+++**	**−**	**−**	**++**	**+++**	**++**	**++**	**−**	**+**	**+**	**+++**
	2191	**+**	**++**	**−**	**−**	**++**	**++**	**++**	**+++**	**+**	**+**	**++**	**+++**	**++**	**++**	**+**	**+**	**++**	**+++**
	2192	**++**	**++**	**++**	**+++**	**++**	**+++**	**++**	**++**	**++**	**+++**	**++**	**+++**	**++**	**++**	**+**	**+**	**++**	**+++**
**D Sal-Ag**	2236	**++**	**++**	**−**	**−**	**−**	**−**	**+**	**+**	**−**	**−**	**+**	**+**	**−**	**+**	**+**	**+**	**++**	**+++**
	2237	**−**	**+**	**−**	**+**	**++**	**++**	**++**	**+++**	**++**	**++**	**++**	**++**	**+**	**+**	**++**	**++**	**+**	**+**
	2238	**+**	**+**	**+**	**+**	**−**	**−**	**−**	**−**	**−**	**+**	**−**	**+**	**++**	**++**	**++**	**+++**	**++**	**++**
	2239	**++**	**++**	**−**	**−**	**++**	**++**	**++**	**++**	**−**	**+**	**+**	**+**	**++**	**++**	**−**	**+**	**+**	**+++**
	2241	**−**	**+**	**−**	**−**	**+**	**++**	**++**	**++**	**−**	**−**	**++**	**++**	**++**	**++**	**++**	**++**	**+**	**+**
	2243	**++**	**++**	**+**	**+**	**+**	**+**	**++**	**++**	**++**	**+++**	**+**	**+**	**++**	**++**	**+**	**+**	**++**	**++**

MLN-mesenteric lymph nodes, ICLN-ileocecal lymph node, Dd-descending duodenum, J-jejunum, IP- ileum proximal, IM-ileum middle, ID-ileum distal, ICO- ileocecal orifice and C-Cecum. CT- indicates culture. – colonies/MAP not detected, + <50 CFU, ++ >50CFU, +++ >300 CFU.

**Table 4:** MAP fecal shedding post immunization and challenge.

Groups	Goat ID	Weeks								
		0wk	3wk	6wk	10wk	14wk	18wk	22wk	26wk	30wk
**A PBS**	2194	**−**	**−**	**−**	**−**	**++**	**−**	**+++**	**++**	**+**
	2198	**−**	**−**	**−**	**−**	**−**	**++**	**+**	**−**	**−**
	2199	**−**	**−**	**−**	**−**	**−**	**−**	**+**	**+**	**−**
	2200	**−**	**−**	**−**	**−**	**++**	**−**	**+++**	**++**	**++**
	2201	**−**	**−**	**−**	**−**	**+**	**+**	**+**	**−**	**++**
	2202	**−**	**−**	**−**	**−**	**++**	**+**	**+++**	**++**	**+++**
**B MAP316F**	2220	**−**	**−**	**−**	**−**	**+**	**−**	**−**	**−**	**+**
	2222	**−**	**−**	**−**	**−**	**−**	**−**	**−**	**+**	**−**
	2224	**−**	**−**	**−**	**−**	**−**	**−**	**+**	**−**	**+**
	2226	**−**	**−**	**−**	**−**	**−**	**−**	**−**	**+**	**−**
	2230	**−**	**−**	**−**	**−**	**−**	**+**	**−**	**−**	**+**
	2234	**−**	**−**	**−**	**−**	**−**	**−**	**−**	**+**	**−**
**C Sal-Vector**	2183	**−**	**−**	**−**	**−**	**+**	**−**	**−**	**−**	**+**
	2184	**−**	**−**	**−**	**−**	**+**	**−**	**+**	**−**	**−**
	2189	**−**	**−**	**−**	**−**	**−**	**−**	**+**	**−**	**−**
	2190	**−**	**−**	**−**	**−**	**−**	**+**	**−**	**+**	**++**
	2191	**−**	**−**	**−**	**−**	**−**	**+**	**−**	**−**	**+**
	2192	**−**	**−**	**−**	**−**	**−**	**++**	**−**	**−**	**++**
**D Sal-Ag**	2236	**−**	**−**	**−**	**−**	**+**	**−**	**−**	**−**	**+**
	2237	**−**	**−**	**−**	**−**	**+**	**−**	**−**	**+**	**−**
	2238	**−**	**−**	**−**	**−**	**−**	**−**	**++**	**−**	**−**
	2239	**−**	**−**	**−**	**−**	**−**	**+**	**−**	**+**	**−**
	2241	**−**	**−**	**−**	**−**	**−**	**+**	**−**	**−**	**−**
	2243	**−**	**−**	**−**	**−**	**−**	**−**	**++**	**−**	**−**

– indicates colonies/MAP not detected, + <50 CFU, ++ >50CFU, +++ >300 CFU.

## Discussion

Live attenuated *Salmonella* present a very versatile tool to deliver an antigen into the cytosol via their type III secretion system (T3SS) which is prerequisite for eliciting a CMI required to control MAP infection in the relevant host. Several investigators have successfully exploited this system to elicit protective immunity against various infections by inducing mucosal, humoral and CMI responses against heterogenous antigens [33], [34]. Recently attenuated *Salmonella* expressing *Mycobacterium tuberculosis* (M tb) antigens induced significant protection against challenge with virulent M. tb H37Rv, which was equivalent to or better than the protection achieved with BCG [35], [36]. Since MAP share similar morphological and pathological features with M. tb, it is tempting to exploit T3SS of *Salmonella* to express and secrete MAP antigens in order to develop an effective vaccine against Johne's disease in cattle and other ruminants. In a previous study, we demonstrated the protective potential of attenuated *Salmonella* expressing a fusion product of MAP protective antigens (Sal-Ag) against challenge in mice. Encouraged by these results, we tested this vaccine (Sal-Ag) in goats (ruminant), which are a natural host. We excluded antigen 74F in the current study as T3SS of *Salmonella* failed to secrete full length which forced us to truncate the antigen. The partial 74F fragment was not able to significantly enhance the protective efficacy of the vaccine. Although PBMCs of Sal-Ag induced significantly lower level of IFN-γ than MAP316F, the level was significantly higher than PBS and vector controls at week 6, 10 and 14 which correlated well with partial protection as increased expression of this cytokine is observed during early stage of infection and reduction in its level is the hallmark of clinical JD [37], [38]. The low level of antibodies induced after vaccination, which declined sharply after challenge, agrees well with previous reports where strong humoral responses occur at a later time point after infection and suggests that antibodies are usually not protective but is indicative of breakdown of CMI leading to rapid progression of disease [39], [40], [40,41]. Significant differences in proliferation and activation of T cells (CD4 and CD8) were observed between Sal-Ag and MAP316F correlating to protection, which is in accordance with previous studies that showed that cows infected with MAP have increased levels of these cells [40], [42]. Partial protection observed in animals immunized with Sal-Ag may be attributed to production of low levels of Th1 cytokines that are important for proper functioning and maintenance of T cells and activation of macrophages for bactericidal activity (Fig. 5A). Although Sal-Ag induced Th2 cytokines, their level was low and similar to the level of Th1 cytokines at earlier time points indicating that the response that was generated was not biased, which is contradictory to previous reports where delivery of antigens through T3SS induced a biased Th1 response. It is likely that the fusion antigen secreted by attenuated *Salmonella* into the cytosol was not only processed and presented by MHC I molecules but was also presented by MHC II molecules due to targeting to phagosomes leading to activation of CD4 T cells (Fig. 5C). Similar to previous studies the current study has shown subsets of T cells producing IFN-γ, IL-17, and IL-22 are involved in the immune response to MAP [43]. The sharp decline in the level of IL-17 8 weeks post challenge in all groups indicates that IL-2 might be suppressing this cytokine [44].

In spite of the generation of proinflammatory cytokines, we did not observe any histopathological lesions in any of the tissues analyzed in either the control or vaccinated groups, probably due to the fact that these animals were kept for only a short period (6 months) after challenge. It would have been interesting to see if any lesions developed with corresponding changes in the immune profile at later time points if we could have kept these animals longer. The data of the present study not only correlate to the observations made in the previous studies by *Stewart et. al*. but also provide detailed insight to early immune responses (antibody, IFN-γ, cytokines, T cells) to MAP vaccination and infection [39], [41]. Most importantly, our data indicate that Sal-Ag provided partial protection as it was able to limit MAP colonization to some extent and the tissue burden was low to moderate but did not achieve a statistically significant level that was seen with MAP316F. We speculate that this is due to failure of T3SS of *Salmonella* to express and secrete full length MAP protective antigens which compelled us to use only the partial fragments that could be secreted for immunization. These partial fragments might be devoid of some of the T cell epitopes required to elicit a protective immune response and thus may account for reduced protective efficacy observed in the current study. Moreover, the fact that Sal-Ag induced significant protection in mice but imparted only partial protection in goats indicates that the way the antigen is processed or presented to the immune system of the host might contribute to suppressing the immune response or *Salmonella* might have failed to express or secrete the optimum level of fusion antigen necessary to generate a protective immune response in goats.

In conclusion, attenuated *Salmonella* expressing heterologous antigen holds high promise in development of cost effective veterinary vaccines and this system may perform very well in case of other infectious and zoonotic diseases.

## Supporting Information

Figure S1 **Raw data for lymhoproliferation, IFN-γ response, Cytokines and FACS analysis.**
(XLS)
